# Evolving Trends and Core Contributors: A Bibliometric Update on Liposomal Bupivacaine

**DOI:** 10.1155/prm/9222122

**Published:** 2026-08-20

**Authors:** Wenwen Zhai, Zhuoying Yu, Xinru Zhao, Huili Liu, Min Li

**Affiliations:** ^1^ Department of Anesthesiology, Peking University Third Hospital, No. 49 North Garden Road Haidian District, Beijing 100191, China, puh3.net.cn

**Keywords:** analgesia, bibliometric update, CiteSpace, liposomal bupivacaine, VOSviewer

## Abstract

**Purpose:**

This study aims to examine the academic output, key research hotspots, collaborative networks, and trends in liposomal bupivacaine research using bibliometric methods. By providing a broad overview, we seek to clarify the knowledge structure and emerging trends in its clinical applications in perioperative analgesia.

**Methods:**

We searched the Web of Science Core Collection database on November 4, 2025, to find relevant articles and reviews. Analytical tools such as CiteSpace and VOSviewer were used to evaluate publication years, countries/regions, institutions, journals, highly cited literature, terms, collaboration networks, and cocitation clusters.

**Results:**

The dataset comprised 1,069 valid publications from 101 countries/regions, involving 3,256 institutions and 13,736 authors. The annual publication output grew slowly before 2015, increased rapidly after 2016, and reached a peak in 2021. The United States (US) was the leading contributor with 775 publications, followed by China with 155 publications. The Harvard University System and the University of Texas System were identified as central research institutions. *Journal of Arthroplasty* published the largest number of articles, while *Anesthesia and Analgesia* recorded the highest average citations per article. Term co‐occurrence and burst analyses identified four major research clusters: formulation and pharmacological foundations, safety considerations, procedure‐specific analgesia, and emerging regional anesthesia techniques. Recent research hotspots included erector spinae plane block, thoracic surgery, and paravertebral block, indicating a shift toward novel nerve block applications and complex surgical scenarios.

**Conclusion:**

Research on liposomal bupivacaine has evolved from formulation development to a clinically oriented field with strong interdisciplinary integration and expanding procedural applications. The US remained the leading contributor to global research and collaboration. These findings provide a structured overview of the evolving evidence base and might help guide future procedure‐specific research and perioperative analgesia strategies.

## 1. Introduction

Effective postoperative analgesia requires the duration of analgesic coverage to match the period of peak postoperative pain. However, single‐injection conventional local anesthetics, such as bupivacaine hydrochloride and ropivacaine, often provide analgesia for less than 24 h [1], which may be insufficient for procedures associated with prolonged moderate‐to‐severe postoperative pain [2].

To address this duration gap, two primary approaches have been used in clinical practice. Continuous peripheral nerve blocks and continuous local infiltrations prolong analgesia duration, but they are associated with catheter‐related infection, local anesthetic systemic toxicity, and increased procedural complexity [3]. Alternatively, adjuvants such as dexamethasone can modestly extend the duration of traditional local anesthetics; however, the magnitude of prolongation is limited, rebound pain may occur after block resolution, and opioid consumption may subsequently increase [4].

Liposomal bupivacaine was developed to address these problems. As the first sustained‐release local anesthetic approved by the United States (US) Food and Drug Administration (FDA), this formulation employs a phospholipid bilayer encapsulation technique to deliver biocompatible and biodegradable spherical lipid particles, measuring 10–30 μm [5]. This design facilitates sustained drug release over 48–72 h [6], with the goal of better covering the typical peak period of postoperative pain and fulfilling the primary objectives of enhanced recovery after surgery (ERAS) [7]. Since its approval for surgical wound infiltration in 2011 [8], its clinical use has expanded to interscalene brachial plexus, femoral, popliteal/sciatic nerve blocks, and fascial plane blocks [9–12]. Theoretically, compared to conventional bupivacaine, liposomal bupivacaine may offer potential advantages such as reduced systemic exposure and improved tissue targeting, making it a widely discussed option for multimodal analgesia [13, 14]. However, the clinical literature remains controversial. Although numerous studies have evaluated liposomal bupivacaine in orthopedic [1, 14], general surgical [7], and thoracic surgical settings [15], its effects on postoperative pain scores, opioid consumption, length of hospitalization, and healthcare costs remain inconsistent [12, 16–18]. Several rigorous trials and meta‐analyses have reported inconsistent or modest clinical benefits, suggesting that its superiority over conventional local anesthetics has not been definitively established in all surgical scenarios [12, 16, 19]. Current evidence remains fragmented and interdisciplinary, spanning multiple surgical specialties, study designs, and clinical settings. While systematic reviews can assess its efficacy, they are less able to characterize the overall research landscape, evolution patterns, and knowledge structures from a macroscopic perspective.

Bibliometrics refers to the quantitative analysis of academic literature, employing statistical methods to map publishing trends and reveal structural patterns within the scholarly corpus [20]. For example, a bibliometric investigation of sugammadex, a selective relaxant‐binding agent, mapped its developmental trajectory from basic pharmacological principles to specific patient applications, while highlighting key areas such as pharmacokinetics and clinical advancements [21].

To address a comparable gap in the literature, this study utilized bibliometric analysis to provide an objective and visual representation of the research landscape surrounding liposomal bupivacaine. Specifically, we used the Web of Science Core Collection (WoSCC) database together with CiteSpace and VOSviewer to perform a comprehensive bibliometric analysis of liposomal bupivacaine research. Our aim is to map the global research patterns, identify key contributors, and delineate emerging trends in liposomal bupivacaine research. The findings may provide a structured reference for clinical practice and help guide future studies in this field.

## 2. Methods

### 2.1. Search Strategy and Data Collection

We conducted a literature search on November 4, 2025, using the Science Citation Index Expanded (SCI‐Expanded) within the WoSCC. Our search targeted publications concerning liposomal bupivacaine, employing the following terms: TS = (liposomal bupivacaine) OR (bupivacaine liposome) OR (Exparel) OR (liposome‐formulated bupivacaine) OR (bupivacaine lipid formulation). No date restriction was applied during the initial search. After retrieval, records were screened stepwise by document type, language, and topic relevance. Only records indexed as “Article” or “Review Article” in WoSCC were retained for further evaluation. The initial search results were downloaded from WoSCC as plain text files, including full records and cited references. Subsequently, WZ and HL independently reviewed the titles and abstracts of all retrieved results to exclude studies not pertinent to liposomal bupivacaine, such as those focusing on alternative local anesthetics or nonliposomal bupivacaine formulations. Discrepancies were resolved through discussion. The inclusion criteria were as follows [1]: studies where liposomal bupivacaine (or its recognized formulations) was the primary subject of investigation [2]; both clinical (e.g., clinical trials, observational studies) and preclinical (e.g., pharmacological, animal models) studies were included; and [3] systematic reviews or meta‐analyses focusing on its efficacy, safety, pharmacology, or clinical application of liposomal bupivacaine. The exclusion criteria were as follows [1]: studies primarily focusing on other local anesthetics where liposomal bupivacaine was merely mentioned as background [2]; studies of nonliposomal extended‐release formulations [3]; document types other than articles or reviews (e.g., meeting abstract, letter, editorial material, note, news item, correction, book chapter, reprint, correction addition, and retracted publication) [4]; non‐English publications.

### 2.2. Data Extraction and Bibliometric Analysis

We systematically extracted the following bibliometric metrics from the retrieved records: publication year, title, authors, affiliations, countries/regions, source journal, journal impact factor, total citation count, and abstract. Terms were subsequently extracted from article titles and abstracts. We merged author names with different abbreviations and writing styles to prevent duplicate statistics and unified all journal titles in accordance with official JCR nomenclature. Institutional names and country information were analyzed according to the Web of Science institutional fields and aggregation labels to ensure reproducibility of the bibliometric results. Prior to VOSviewer analysis, synonymous expressions were merged using a thesaurus file to reduce redundancy and improve visualization consistency. For example, “liposome bupivacaine,” “bupivacaine liposome,” and related variants were standardized under the displayed label “liposomal bupivacaine” in the visualization. Vague, overly general, or noninformative terms were removed. Terms appearing fewer than 10 times were excluded to improve visualization clarity. The data were organized and descriptively analyzed using Microsoft Excel (Microsoft Corporation, Redmond, WA, USA). Bibliometric analyses were performed using the online bibliometric platform bibliometric.com (https://bibliometric.com), VOSviewer (Version 1.6.20; Centre for Science and Technology Studies, Leiden University, Leiden, the Netherlands), and CiteSpace (Version 6.4.R1; Chaomei Chen, Drexel University, Philadelphia, PA, USA). The online platform was used to generate the country collaboration chord diagram. VOSviewer was used for coauthorship, cocitation, and term co‐occurrence visualizations using the full counting method. The normalization method was association strength, with attraction = 2, repulsion = 0, clustering resolution = 1.00, and minimum cluster size = 10. For term co‐occurrence analysis, the minimum occurrence threshold was set at 10. For journal cocitation and reference cocitation analyses, the minimum citation threshold was set at 20. CiteSpace was used for burst detection analysis and related visualization with the following settings: time slicing = 1992–2025, years per slice = 1, node type = Keyword, selection criteria = g‐index (*k* = 25), and pruning = Pathfinder.

### 2.3. Ethics Approval

This study did not involve human participants, animals, or identifiable personal data. Since the analysis was conducted exclusively using publicly available bibliographic records from the WoSCC, ethical approval and informed consent were not applicable for this research.

## 3. Results

### 3.1. Overall Scale Analysis of the Data

**FIGURE 1:**
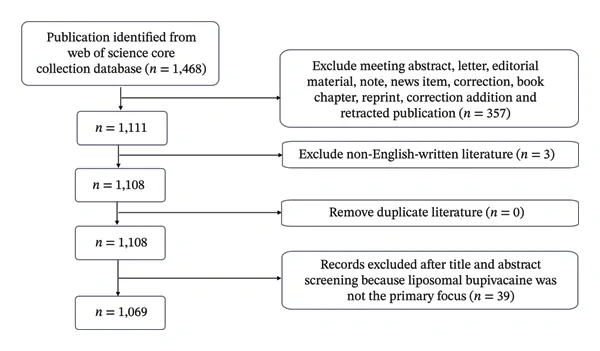
Flowchart of literature search.

The first search yielded 1,468 records. After excluding 357 records with document types other than Article or Review Article (meeting abstract, letter, editorial material, note, news item, correction, book chapter, reprint, correction addition, and retracted publication) and 3 non‐English papers (no duplicates), 1108 records underwent title and abstract screening. Subsequently, 39 studies were excluded because liposomal bupivacaine was not the primary focus. Finally, 1,069 papers originating from 101 countries/regions, involving 3,256 institutions and 13,736 authors, were included in the final analysis (Figure 1).

### 3.2. Annual Publication Trends

**FIGURE 2:**
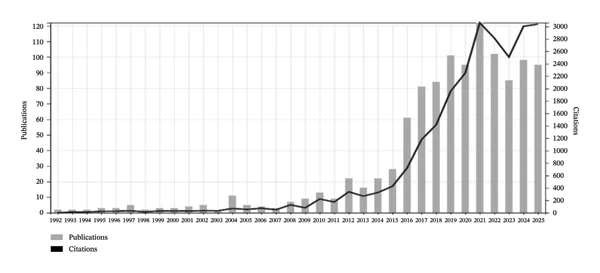
Annual publication and citation counts.

The annual number of published papers showed an upward trend, which could be divided into two phases: a slow growth prior to 2015, followed by a rapid increase from 2016 to 2025, reaching a peak in 2021 (Figure 2). Because the literature search was conducted on November 4, 2025, publication output for 2025 should be interpreted with caution.

### 3.3. Country‐Level Publication Distribution

**FIGURE 3:**
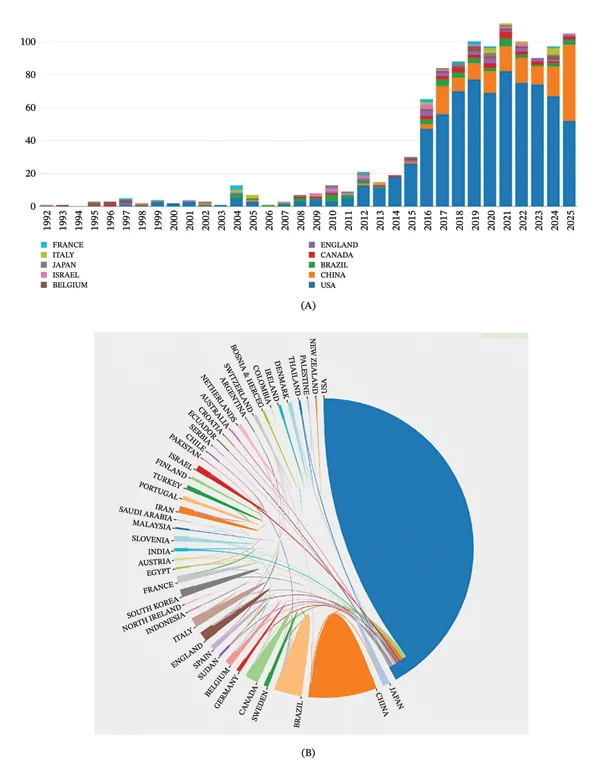
Country contributions and collaboration networks. (A) Annual distribution of publications for the top 10 countries by publication volume. (B) Country collaboration chord diagram, where the sector area corresponds to each country’s publication volume, and the connecting lines (with thickness denoting collaboration intensity) represent cross‐national research partnerships.

Substantial differences were observed in the number of publications on liposomal bupivacaine across countries (Table 1). The US had the largest output, with 775 publications (72.5%), the highest number of citations (18,714), and the highest H‐index (67). China ranked second, with 155 publications and an average citation rate of 10.62 per paper. Some countries with fewer publications had relatively high citation impact, including Israel (47.70 average citations per article), England (39.25), and Canada (38.83). Figure 3A shows the number of publications for the top 10 countries.

**TABLE 1:** Country‐level publication output.

Rank	Country	Paper count	Total citations	H‐index	Average citations
1	United States	775	18,714	67	24.15
2	China	155	1646	22	10.62
3	Brazil	46	901	19	19.59
4	Canada	29	1126	16	38.83
5	Belgium	22	788	15	35.82
6	Israel	20	954	15	47.70
6	England	20	785	13	39.25
8	Japan	16	542	12	33.88
9	Italy	14	299	10	21.36
10	France	13	380	10	29.23

The international collaboration network is shown in the chord diagram (Figure 3B), corresponding to the quantitative measure presented in Table 1. The US was the main hub and had strong collaborations with other leading countries such as China, Japan, Canada, and England. In contrast, although China ranked second in publication volume, its collaborative network was more concentrated, primarily involving the US and Brazil. Several other countries, including Turkey, Portugal, and Egypt, displayed limited international connections, suggesting relatively isolated research output.

Countries with high average citation rates (Canada 38.83, England 39.25, and Israel 47.70) also showed stronger international links with the US. By contrast, China had a lower average citation rate (10.62) and a comparatively less extensive international collaboration network. These patterns were observed concurrently, but no causal relationship can be inferred from the present bibliometric analysis.

### 3.4. Institutional Analysis

Institutional analysis showed that liposomal bupivacaine research was predominantly led by institutions in the US, with Brazil also contributing prominently among the top‐ranked organizations. After consolidating affiliated Harvard entities into the Harvard University System, this institution ranked first with 146 publications, followed by the University of Texas System with 54 publications. Mayo Clinic, the University of California System, and Cleveland Clinic Foundation were also among the leading contributors, with 45, 43, and 36 publications, respectively. Among non‐US institutions, Universidade Estadual de Campinas (37 publications) and Universidade de São Paulo (29 publications) were the only institutions in the top 10, highlighting Brazil’s notable contribution to this field. In terms of citation impact, Thomas Jefferson University had the highest average citations per article (48.44), followed by the Harvard University System (39.32). Overall, these findings indicated that institutional productivity was highly concentrated in the US, while selected Brazilian universities also played an important role in the global research landscape of liposomal bupivacaine (Table 2).

**TABLE 2:** Top 10 institutions.

Rank	Institution	Country	Records	Total citations	H‐index	Average citations per article
1	Harvard University System	United States	146	5741	27	39.32
2	University of Texas System	United States	54	1697	24	31.43
3	Mayo Clinic	United States	45	1091	19	24.24
4	University of California System	United States	43	1277	20	29.70
5	Universidade Estadual de Campinas	Brazil	37	798	17	21.57
6	Cleveland Clinic Foundation	United States	36	796	17	22.11
7	Universidade de São Paulo	Brazil	29	742	18	25.59
8	University System of Ohio	United States	27	814	17	30.15
9	Duke University	United States	26	665	15	25.58
10	Thomas Jefferson University	United States	25	1211	18	48.44

### 3.5. Journal Analysis

Research on liposomal bupivacaine was published mainly in orthopedic, anesthesiology, pain medicine, and pharmaceutical journals. The *Journal of Arthroplasty* published the largest number of articles, with 42 publications, 1,646 citations, and an H‐index of 25. *Journal of Shoulder and Elbow Surgery* published 35 articles, and *Regional Anesthesia and Pain Medicine* published 24 articles. These rankings highlight the importance of joint surgery and regional anesthesia in this field. Among the top 10 journals, *Anesthesia and Analgesia* had the highest average citation rate, with 49.05 citations per article, followed by *Anesthesiology* with 46.15 citations per article. *International Journal of Pharmaceutics* ranked ninth by publication volume, with 15 articles. The top 10 journals are listed in Table 3.

**TABLE 3:** Top 10 journals by publication count.

Rank	Journals	Paper count	Total citations	H‐index	IF (2024)/JCR
1	Journal of Arthroplasty	42	1646	25	3.8/Q1
2	Journal of Shoulder and Elbow Surgery	35	671	16	2.9/Q1
3	Regional Anesthesia and Pain Medicine	24	876	13	3.5/Q1
4	Anesthesia and Analgesia	21	1030	17	4.0/Q1
4	Plastic and Reconstructive Surgery	21	645	12	3.4/Q1
6	Anesthesiology	20	923	16	9.2/Q1
7	Journal of Pain Research	19	335	12	2.5/Q2
8	Journal of Clinical Anesthesia	18	262	9	5.1/Q1
9	Journal of Bone and Joint Surgery	15	451	10	4.4/Q1
9	International Journal of Pharmaceutics	15	217	9	5.2/Q1

**FIGURE 4:**
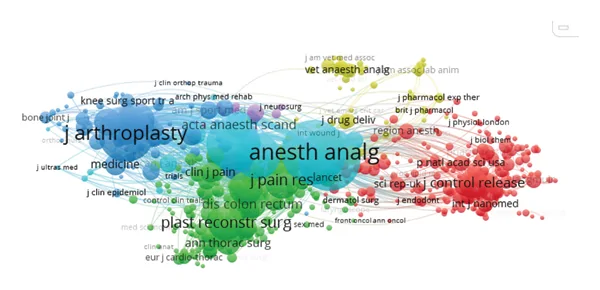
Journal cocitation clustering map. Each node represents a journal; node size corresponds to cocitation frequency (reflecting the journal’s influence in the field), node color denotes thematic clusters, and line thickness indicates cocitation intensity (the frequency with which two journals are cited together in the literature).

Figure 4 shows four main discipline‐aligned clusters which correspond broadly to the journals listed in Table 3. The blue cluster consisted mainly of orthopedic journals, such as the *Journal of Shoulder and Elbow Surgery* and the *Journal of Bone and Joint Surgery*. The large node size of *Journal of Arthroplasty* corresponded to its high publication volume and reflected the close association between liposomal bupivacaine research and joint surgery. The cyan cluster included anesthesiology journals, such as *Anesthesia and Analgesia* and *Regional Anesthesia and Pain Medicine*. The dense links within this cluster reflected the concentration of regional analgesia research in high‐impact anesthesiology outlets. The green cluster contained surgical and pain medicine journals, such as *Plastic and Reconstructive Surgery and Journal of Pain Research*, and mainly reflected the application of liposomal bupivacaine in nonorthopedic surgical analgesia and general postoperative pain management. The red cluster was dominated by pharmaceutical journals, including the *Journal of Controlled Release and International Journal of Pharmaceutics*. Notably, a strong cocitation relationship was observed between the orthopedic and anesthesiology clusters, indicating frequent cross‐disciplinary knowledge exchange. One small yellow cluster, including journals such as *Veterinary Anesthesia and Analgesia*, represented nonhuman research and showed limited cocitation links with the core clinical clusters.

### 3.6. High‐Impact Cited References and Cocitation Clustering

We ranked the top 20 high‐impact cited references within the liposomal bupivacaine research corpus based on citation frequency (Supporting Table S1). The total number of citations ranged from 125 to 287, with an average of 176 citations. These papers were published between 2004 and 2022, with 16 of the top 20 publications (80%) appearing between 2011 and 2018.

These highly cited references mainly reflected foundational research, pharmacology, clinical applications, and evidence‐based evaluations. Foundational research and pharmacology focused on optimizing liposomal drug‐loading efficiency based on loading conditions and drug physicochemical properties [6, 22, 23]. Clinical applications covered a wide range of procedure‐specific postoperative analgesic regimens, prominently featuring hemorrhoidectomy [24], joint replacement (such as total knee arthroplasty) [8, 25–27], hallux valgus correction [28], and spinal surgery [29–31]. Evidence‐based evaluations synthesized clinical safety and efficacy, particularly local anesthetic systemic toxicity [32] and the comparative effectiveness of continuous nerve blocks [33]. These publications appeared in leading journals in anesthesiology, orthopedics, and pharmacy.

**FIGURE 5:**
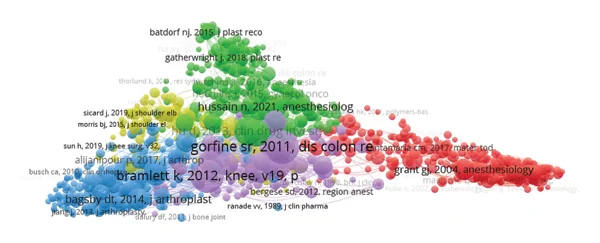
Reference cocitation clustering map. Each node represents a cited reference, node size corresponds to total citation frequency, node color denotes distinct thematic clusters, and line thickness reflects cocitation intensity.

Figure 5 reveals five clusters. The purple cluster represented foundational and pharmacological research, with Zucker et al. [23] and Grant et al. [6] as central nodes. The blue cluster in the lower left region focused on joint arthroplasty research, with Bramlett et al. [8] as the core node. The red cluster on the right encompassed evidence‐based evaluation and regional blocks, with Neal et al. [32] and Hussain et al. [16] as core nodes. The green and yellow clusters covered applications beyond joint arthroplasty: the green cluster featured general surgical procedures such as hemorrhoidectomy (Gorfine et al.) [24], while the yellow cluster was related to other orthopedic surgeries. Thick links between the purple and blue clusters indicated frequent cocitation between foundational research and clinical arthroplasty trial literature.

### 3.7. Term Analysis

**FIGURE 6:**
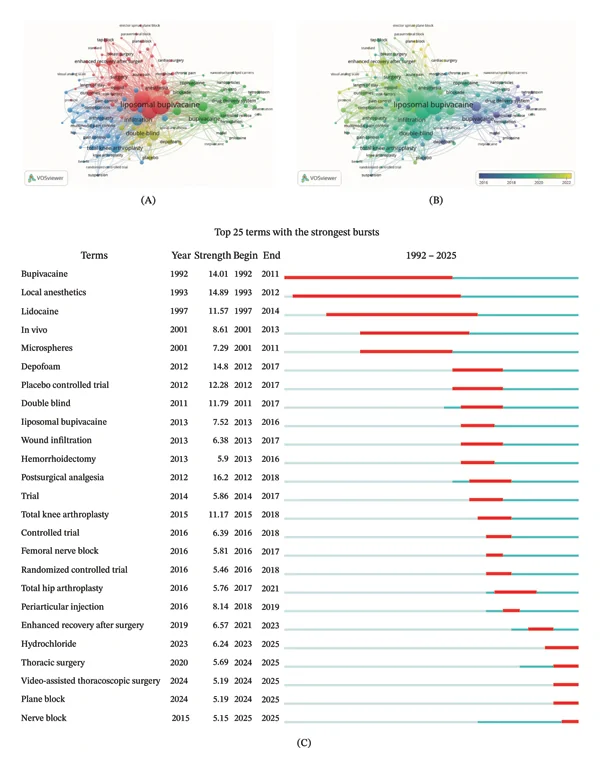
Term co‐occurrence, overlay, and burst analysis. (A) Term distribution map showing 144 terms grouped into four distinct clusters. (B) Overlay visualization of terms by average publication year, where purple nodes represent earlier publication years and yellow nodes denote more recent publication years. (C) Burst detection analysis of the top 25 terms with the strongest bursts generated by CiteSpace. In panels A and B, each node represents a term, with its size proportional to occurrence frequency; shorter distances between nodes indicate stronger co‐occurrence relationships. Note: Burst detection in panel C was performed in CiteSpace using “keyword” as the node type, which is a software parameter. Because the analyzed items were derived from titles and abstracts, they are described as terms throughout the manuscript.

Terms were defined as words or phrases appearing at least 10 times in article titles and abstracts, resulting in 144 terms. In the VOSviewer term co‐occurrence map (Figure 6A), the most frequent term was the standardized label “liposomal bupivacaine,” with 668 occurrences and a link strength of 4,407, followed by “Analgesia” with 485 occurrences and a link strength of 3,379, “Pain” with 471 occurrences and a link strength of 3,099, “Infiltration” with 240 occurrences and a link strength of 1,919, and “Bupivacaine” with 224 occurrences and a link strength of 1,397.

The 144 terms were grouped into four clusters: Cluster 1 (green, 41 terms) represented pharmacological properties; Cluster 2 (yellow, 13 terms) represented safety and side effects; Cluster 3 (blue, 39 terms) and Cluster 4 (red, 51 terms) represented clinical application and analgesia. Cluster 3 mainly focused on orthopedic procedures, such as hip and knee arthroplasty, whereas Cluster 4 emphasized analgesic use in various surgical procedures and nerve block techniques. The most recent terms were mainly located in Cluster 4, indicating the potential expansion of liposomal bupivacaine into new clinical applications.

Figure 6B shows the same co‐occurrence map, with nodes colored according to the average publication year of the terms. Purple nodes indicate earlier publication years, whereas yellow nodes denote more recent publication years. Chronologically, recent terms included “erector spinae plane block,” “thoracic surgery,” “paravertebral block,” and “intercostal nerve block,” suggesting current research hotspots and trends.

Figure 6C presents the burst detection map of the top 25 terms with the strongest bursts generated by CiteSpace. During the early exploratory phase, burst terms mainly included “bupivacaine” from 1992 to 2011, “local anesthetics” from 1993 to 2012, “lidocaine” from 1997 to 2014, as well as “in vivo” from 2001 to 2013 and “microspheres” from 2001 to 2011. Between 2012 and 2018, burst terms such as “depofoam,” “placebo controlled trial,” “double blind,” “postsurgical analgesia,” and “total knee arthroplasty” reflected increasing attention to clinical trials and procedure‐specific analgesia. More recent burst terms included “hydrochloride,” “thoracic surgery,” “video‐assisted thoracoscopic surgery,” “plane block,” and “nerve block,” suggesting growing interest in comparative formulations and regional anesthesia applications in recent publications.

## 4. Discussion

To our knowledge, this study represents one of the first comprehensive bibliometric analyses to systematically map the global research landscape of liposomal bupivacaine.

### 4.1. Global Distribution of Publications and Collaboration Patterns

By integrating publication output, collaboration networks, and citation impact, our findings suggested a highly centralized research structure dominated by the US, accompanied by marked disparities in international contribution and influence. The US accounted for more than two‐thirds of all publications and citations, and this dominance was consistently reflected at both national and institutional levels. This leading position might be associated with several contextual factors, including earlier regulatory approval and broader clinical adoption in the US. Liposomal bupivacaine received US FDA approval in 2011 [17]. In the years that followed, large patient cohorts [34] and multicenter randomized trials [24, 28] were progressively reported. During the same period, research output increased, and the US remained the central hub of global collaboration. However, these temporal patterns should be interpreted descriptively rather than causally.

In contrast, although China ranked second in publication volume, its average citation rate and international connectivity remained relatively limited. This discrepancy may be related to later regulatory approval in China in 2022 and a currently more limited international collaboration network. In addition, several available studies were conducted in single centers [35, 36]. This pattern was observed alongside relatively limited international visibility, although the present analysis cannot determine whether study scale directly influenced citation impact or collaboration patterns. At the same time, late entry into the field may allow emerging research centers to focus directly on pragmatic and large‐scale studies aligned with ERAS protocols and opioid‐sparing strategies [7, 37–39]. Overall, the observed “core‐periphery” collaboration structure reflects an uneven global research landscape. Future efforts should prioritize multicenter, cross‐regional collaboration to promote a more balanced and globally representative evidence base.

### 4.2. Multidisciplinary Framework of Liposomal Bupivacaine Research

The journal cocitation analysis, reference clustering, and term co‐occurrence analysis collectively suggested that liposomal bupivacaine research was inherently multidisciplinary, integrating pharmaceutical sciences, anesthesiology, and surgical specialties, particularly orthopedics. This interdisciplinary structure reflected the translational trajectory of liposomal bupivacaine from formulation development to procedure‐specific clinical application.

From the pharmaceutical perspective, highly cited foundational studies on liposomal formulation and pharmacokinetics established the mechanistic basis for sustained drug release and safety [23]. These studies formed a tightly connected knowledge base and were frequently cocited with subsequent clinical research. In parallel, anesthesiology‐focused journals emphasized regional anesthesia techniques, multimodal analgesia, and safety considerations [40], while orthopedic journals concentrated on procedure‐specific outcomes, particularly in joint arthroplasty [41, 42].

The strong cocitation links among pharmaceutical, anesthesiology, and orthopedic journals suggested close links between formulation research and clinical applications. Knowledge generated from formulation research was frequently cocited with clinical trial literature, while surgical outcome studies were closely linked to literature on anesthetic strategy optimization. This bidirectional knowledge flow underscored the important role of interdisciplinary collaboration in perioperative analgesia research.

### 4.3. Evolution of Research Hotspots and Emerging Directions

Analysis of highly cited references and term burst detection revealed a clear temporal evolution of liposomal bupivacaine research.

Early investigations primarily focused on formulation optimization, pharmacokinetics, and safety, addressing the fundamental challenge of extending analgesic duration beyond that of conventional local anesthetics [43–46]. Following regulatory approval for surgical site infiltration, research emphasis shifted toward procedure‐specific clinical applications, most notably in joint arthroplasty and other standardized surgical models [47–49]. These settings were commonly used to evaluate analgesic efficacy, opioid‐sparing potential, and patient‐centered outcomes.

Subsequent expansion into peripheral nerve blocks reflected a transition from incision‐based analgesia to targeted regional anesthesia [50–52]. More recent terms identified in the overlay, including “erector spinae plane block” and “paravertebral block,” together with recent burst terms such as “thoracic surgery,” indicated a growing interest in fascial plane blocks and complex surgical applications [12, 15, 53]. Notably, these emerging areas appeared in parallel with growing clinical interest in unmet analgesic needs. These patterns suggest a possible research trajectory: from addressing the “duration gap” of traditional local anesthetics to optimizing procedure‐specific analgesia, and ultimately integrating liposomal bupivacaine into ERAS‐based perioperative management strategies. Importantly, bibliometric prominence should not be interpreted as definitive evidence of clinical superiority, underscoring the need for well‐designed, procedure‐specific randomized trials to resolve ongoing controversies.

It was noteworthy that the annual publication volume appeared to have plateaued or slightly decreased after reaching its peak around 2021 (as shown in Figure 2). This trend might reflect a transition in the research lifecycle of liposomal bupivacaine from a period of rapid exploratory trials to a more mature phase of consolidation. Furthermore, several rigorous, high‐profile systematic reviews and meta‐analyses published recently [12, 16, 19] have reported that perineural liposomal bupivacaine may not offer clinically meaningful superiority over nonliposomal bupivacaine for certain peripheral nerve blocks. These publications coincided with a more cautious and selective pattern in subsequent clinical research; however, this observation remains descriptive and hypothesis‐generating. Future studies should focus on well‐defined patient populations, procedure‐specific indications, and clinically meaningful endpoints, where the potential benefits of liposomal bupivacaine could be more clearly evaluated.

### 4.4. Limitations and Future Work

Several limitations should be acknowledged. First, this analysis was based exclusively on the WoSCC. Although the WoSCC provided comprehensive citation data essential for robust bibliometric analysis using tools such as CiteSpace and VOSviewer, reliance on a single database may introduce selection bias. Relevant studies indexed solely in other major biomedical databases, such as PubMed and Scopus, may have been excluded, potentially leading to underrepresentation of some areas of the global scope of clinical research on liposomal bupivacaine. Second, bibliometric methods emphasize quantitative patterns and cannot fully capture qualitative aspects such as study design rigor, patient selection, risk of bias, or long‐term clinical effectiveness. Third, bibliometric trends should not be interpreted as direct evidence of clinical efficacy or safety. We explicitly acknowledge that our dataset cannot confirm direct causal links between national policy, clinical study scale, and research output, further limiting overinterpretation of our findings. The observed trends suggest several potential directions for future research. These include extending research to pediatric populations following recent regulatory approval, conducting large‐scale prospective studies to assess long‐term safety and effectiveness across diverse patient groups, and strengthening international multicenter collaboration to enhance generalizability.

## 5. Conclusion

In summary, liposomal bupivacaine research has evolved from formulation science to a clinically oriented field characterized by multidisciplinary integration and expanding procedural applications. However, bibliometric prominence should not be interpreted as definitive evidence of clinical superiority. Future well‐coordinated, high‐quality, and procedure‐specific studies are needed to better define its role in modern perioperative analgesia.

## Author Contributions

Wenwen Zhai: conceptualization, methodology, data curation, formal analysis, software, and writing–original draft. Zhuoying Yu: conceptualization, data curation, formal analysis, visualization, and writing–original draft. Xinru Zhao: methodology, data curation, formal analysis, and writing–review and editing (substantial revision). Huili Liu: project administration, supervision, validation, writing–review and editing, and correspondence. Min Li: project administration, supervision, validation, writing–review and editing, and correspondence. Wenwen Zhai, Zhuoying Yu, and Xinru Zhao contributed equally to this work.

## Funding

This work was supported by the special fund of the National Clinical Key Specialty Construction Program, P.R. China (2025) and Beijing Zhongkanglian Public Welfare Foundation (ZKLGY‐KYFNDYLH‐001).

## Ethics Statement

This study did not involve human participants, animals, or any other identifiable personal data. Since the analysis was conducted exclusively using publicly available bibliographic records from the WoSCC, ethical approval and informed consent were not applicable for this research.

## Conflicts of Interest

The authors declare no conflicts of interest.

## Supporting Information

Additional supporting information can be found online in the Supporting Information section.

## Supporting information


**Supporting Information** Supporting Table S1. Top 20 most cited references.

## Data Availability

The data that support the findings of this study are available from the corresponding author upon reasonable request.
