# Supplementary material for: Evolving Trends and Core Contributors: A Bibliometric Update on Liposomal Bupivacaine
Source: Pain Res Manag. 2026 Aug 20;2026:9222122. doi: 10.1155/prm/9222122 (PMC13494514; doi:10.1155/prm/9222122)
Supplement: Supplementary file 1 [file PRM-2026-9222122-s001.docx]

**Supplementary Table S1. Top 20 most cited references**

| Rank | Total citation | Article title | Journal | Publication year | First author |
| --- | --- | --- | --- | --- | --- |
| 1 | 287 | Liposome drugs' loading efficiency: A working model based on loading conditions and drug's physicochemical properties | *Journal of Controlled Release* | 2009 | Zucker, D. |
| 2 | 263 | Bupivacaine Extended-Release Liposome Injection for Prolonged Postsurgical Analgesia in Patients Undergoing Hemorrhoidectomy: A Multicenter, Randomized, Double-blind, Placebo-controlled Trial | *Diseases of the Colon & Rectum* | 2011 | Gorfine, SR. |
| 3 | 236 | The Third American Society of Regional Anesthesia and Pain Medicine Practice Advisory on Local Anesthetic Systemic Toxicity: Executive Summary 2017 | *Regional Anesthesia and Pain Medicine* | 2018 | Neal, JM. |
| 4 | 235 | A randomized, double-blind, dose-ranging study comparing wound infiltration of DepoFoam bupivacaine, an extended-release liposomal bupivacaine, to bupivacaine HCl for postsurgical analgesia in total knee arthroplasty | *Knee* | 2012 | Bramlett, K. |
| 5 | 212 | How Much Pain Is Significant? Defining the Minimal Clinically Important Difference for the Visual Analog Scale for Pain After Total Joint Arthroplasty | *Journal of Arthroplasty* | 2018 | Danoff, JR. |
| 6 | 209 | Transversus Abdominis Plane Block: An Updated Review of Anatomy and Techniques | *Biomed Research International* | 2017 | Tsai, HC. |
| 7 | 196 | A Phase 3, Randomized, Placebo-Controlled Trial of DepoFoam® Bupivacaine (Extended-Release Bupivacaine Local Analgesic) in Bunionectomy | *Advances in Therapy* | 2011 | Golf, M. |
| 8 | 193 | Continuous Peripheral Nerve Blocks: An Update of the Published Evidence and Comparison with Novel, Alternative Analgesic Modalities | *Anesthesia and Analgesia* | 2017 | Ilfeld, BM. |
| 9 | 182 | Pharmacokinetic Profile of Liposome Bupivacaine Injection Following a Single Administration at the Surgical Site | *Clinical Drug Investigation* | 2013 | Hu, DD. |
| 10 | 161 | Best evidence in multimodal pain management in spine surgery and means of assessing postoperative pain and functional outcomes | *Journal of Clinical Neuroscience* | 2015 | Devin, CJ. |
| 11 | 145 | Perioperative Pain Management in Hip and Knee Arthroplasty | *Orthopedic Clinics of North America* | 2017 | Gaffney, CJ. |
| 12 | 144 | Development of an Enhanced Recovery After Surgery (ERAS) approach for lumbar spinal fusion | *Journal of Neurosurgery-Spine* | 2017 | Wang, MY. |
| 13 | 143 | Pain management after total knee arthroplasty: PROcedure SPEcific Postoperative Pain ManagemenT recommendations | *European Journal of Anaesthesiology* | 2022 | Lavand'homme, PM. |
| 14 | 140 | Current Strategies in Anesthesia and Analgesia for Total Knee Arthroplasty | *Journal of the American Academy of Orthopaedic Surgeons* | 2016 | Moucha, CS. |
| 15 | 133 | Posterior Intercostal Nerve Block with Liposomal Bupivacaine: An Alternative to Thoracic Epidural Analgesia | *Annals of Thoracic Surgery* | 2015 | Rice, DC. |
| 15 | 133 | A novel liposomal bupivacaine formulation to produce ultralong-acting analgesia | *Anesthesiology* | 2004 | Grant, GJ. |
| 17 | 132 | Endoscopic minimally invasive transforaminal interbody fusion without general anesthesia: initial clinical experience with 1-year follow-up | *Neurosurgical Focus* | 2016 | Wang, MY. |
| 18 | 127 | A Double-Blind, Randomized, Active-Controlled Study for Post-Hemorrhoidectomy Pain Management with Liposome Bupivacaine, a Novel Local Analgesic Formulation | *American Surgeon* | 2012 | Haas, E. |
| 19 | 126 | New Advances in Acute Postoperative Pain Management | *Current Pain and Headache Reports* | 2018 | Mitra, S. |
| 20 | 125 | Perineural Liposomal Bupivacaine Is Not Superior to Nonliposomal Bupivacaine for Peripheral Nerve Block Analgesia: A Systematic Review and Meta-analysis | *Anesthesiology* | 2021 | Hussain, N. |
